# Comparative Efficacy and Safety of P2Y12 Inhibitor Monotherapy Versus Dual Antiplatelet Therapy in Patients Following Percutaneous Coronary Intervention (PCI): A Systematic Review

**DOI:** 10.7759/cureus.88817

**Published:** 2025-07-26

**Authors:** Muhammad Khubaib Ejaz, Mohsin Khan, Nouman Anthony, Saima M Khan, Mihir Nikalje, Marwah Shah, Maleeha Anum, Taj Rajan Jacob Metharayil, Aaqif Sarooj Rasheed, Fathima Ruqaiya Mohmed Amzath

**Affiliations:** 1 Emergency Medicine, Gondal Medical Complex, Gujranwala, Pakistan; 2 Internal Medicine, Rehman Medical Institute, Peshawar, PAK; 3 General Medicine, Rehman Medical Institue, Peshawar, PAK; 4 Internal Medicine, Peshawar Medical College, Peshawar, PAK; 5 Internal Medicine, Walsall Manor Hospital, Walsall, GBR; 6 Medicine, Holy Family Hospital, Dhaka, BGD; 7 Internal Medicine, Jinnah Sindh Medical University, Karachi, PAK; 8 Acute Medicine, Walsall Manor Hospital, Walsall, GBR; 9 General Medicine, Vitebsk State Medical University, Vitebsk, BLR

**Keywords:** bleeding risk, cardiovascular outcomes, clopidogrel, dual antiplatelet therapy, p2y12 inhibitor monotherapy, percutaneous coronary intervention, prasugrel, ticagrelor

## Abstract

Dual antiplatelet therapy (DAPT) is the standard approach for preventing ischemic events following percutaneous coronary intervention (PCI); however, the associated bleeding risk has led to growing interest in P2Y12 inhibitor monotherapy after an initial DAPT period. This systematic review evaluates the efficacy and safety of monotherapy with ticagrelor, clopidogrel, or prasugrel following one to three months of initial DAPT, compared to prolonged DAPT regimens. A comprehensive literature search was conducted in accordance with Preferred Reporting Items for Systematic Reviews and Meta-Analyses (PRISMA) guidelines, identifying randomized controlled trials (RCTs) and high-quality comparative studies published within the last five years. Study quality was assessed using the Cochrane Risk of Bias 2.0 tool for RCTs and the Newcastle-Ottawa Scale (NOS) for post-hoc analyses. Primary outcomes included major adverse cardiovascular events (MACE) and clinically significant bleeding. Monotherapy was associated with a lower incidence of major bleeding (hazard ratio (HR) range: 0.35-0.66) while maintaining comparable ischemic outcomes, with reported MACE HRs ranging from 0.54 to 1.14. Ticagrelor monotherapy showed consistent bleeding reduction, particularly in high-risk populations, whereas clopidogrel demonstrated similar cardiovascular efficacy and a favorable safety profile in select cohorts. Efficacy and safety outcomes varied across studies depending on the agent used and patient characteristics, and moderate between-study heterogeneity was observed in trial design, populations, and outcome definitions. These findings support the use of individualized antiplatelet strategies, with P2Y12 inhibitor monotherapy emerging as a viable option for reducing bleeding risk without compromising ischemic protection in appropriately selected post-PCI patients.

## Introduction and background

Percutaneous coronary intervention (PCI) has revolutionized the management of coronary artery disease (CAD), significantly improving patient outcomes by restoring coronary blood flow [1]. However, post-PCI management remains a critical area of research, particularly in optimizing antiplatelet therapy to balance the risk of ischemic events against bleeding complications. Dual antiplatelet therapy (DAPT), which typically consists of aspirin and a P2Y12 inhibitor such as clopidogrel, ticagrelor, or prasugrel, has long been the standard of care in preventing stent thrombosis and major adverse cardiovascular events (MACE) [2,3]. However, prolonged DAPT use is associated with an increased risk of major bleeding, prompting an ongoing debate on whether a de-escalation to monotherapy after a short-term DAPT regimen might be a safer and equally effective alternative [4].

Several randomized controlled trials (RCTs) have evaluated the efficacy and safety of DAPT compared to monotherapy in post-PCI patients, with varying results depending on the choice of antiplatelet agents, patient risk stratification, and the duration of therapy. While DAPT provides robust ischemic protection [5], emerging evidence suggests that monotherapy with a P2Y12 inhibitor such as ticagrelor or clopidogrel may offer comparable efficacy while reducing bleeding risks, especially in high-risk populations [6]. Given these evolving insights, it is essential to systematically review the latest clinical evidence to determine whether monotherapy after an initial DAPT period provides an optimal balance of safety and efficacy. This systematic review aims to synthesize findings from recent high-quality clinical trials to clarify the comparative effectiveness of DAPT versus monotherapy in patients following PCI, considering ischemic protection, bleeding risk, and long-term cardiovascular outcomes.

The research question for this systematic review is framed using the Population, Intervention, Comparison, Outcome (PICO) [7] model to ensure a structured and evidence-based approach. In post-PCI patients, the choice between DAPT and monotherapy is a crucial determinant of long-term cardiovascular outcomes. The population of interest included patients who have undergone PCI, irrespective of whether they presented with acute coronary syndrome (ACS) or stable CAD. The intervention under evaluation is the use of dual antiplatelet therapy consisting of aspirin combined with a P2Y12 inhibitor for varying durations post PCI. The comparison group consisted of patients receiving monotherapy with either aspirin or a P2Y12 inhibitor after an initial DAPT period. The primary outcomes assessed include MACE, defined as a composite of myocardial infarction (MI), stroke, stent thrombosis, and cardiovascular mortality, alongside bleeding complications such as major bleeding, as classified by the Bleeding Academic Research Consortium (BARC) criteria [8]. This systematic review seeks to determine whether monotherapy following an initial DAPT period provides equivalent ischemic protection while reducing bleeding risks compared to prolonged DAPT in patients following PCI.

## Review

Materials and methods

Search Strategy

The search strategy for this study was meticulously designed to ensure a comprehensive and systematic identification of relevant literature, adhering to the Preferred Reporting Items for Systematic Reviews and Meta-Analyses (PRISMA) guidelines [9]. A structured search was conducted across PubMed, Excerpta Medica database (EMBASE), and the Cochrane Library using predefined Medical Subject Headings (MeSH) terms and keywords related to DAPT, P2Y12 inhibitor monotherapy, PCI, and bleeding outcomes. Boolean operators were employed to refine results and ensure the inclusion of RCTs and high-quality comparative studies. The search was limited to English-language articles published between January 1, 2019, and January 15, 2024, to reflect the most recent and clinically relevant evidence. Reference lists of key articles were also manually screened to identify any additional eligible studies.

Titles and abstracts were independently screened by multiple reviewers, followed by full-text assessment using predefined inclusion and exclusion criteria. Any discrepancies were resolved through discussion or consultation with a third reviewer to ensure consistency and minimize bias. While the review methodology was aligned with PRISMA guidelines, the study was not registered in the International Prospective Register of Systematic Reviews (PROSPERO) due to time constraints and the rapid initiation of the data collection process following the development of the protocol. However, we maintained transparency and methodological rigor through detailed documentation of eligibility criteria, data extraction procedures, and quality assessment tools, all of which are presented within this manuscript to ensure reproducibility and credibility.

Eligibility Criteria

The eligibility criteria for this systematic review were designed to ensure the inclusion of high-quality, relevant studies that directly compared DAPT with P2Y12 inhibitor monotherapy in patients undergoing PCI. Study selection was guided by the PICO framework to align with the research question. We included only RCTs and comparative post-hoc analyses derived from RCT data, both of which involve predefined control and intervention groups, providing robust comparative effectiveness data. No observational, registry-based, or retrospective studies were included to maintain methodological rigor. The target population included adult patients with ACS or stable ischemic heart disease who had undergone PCI with drug-eluting stent implantation. To ensure meaningful assessment of outcomes, studies were required to have a minimum follow-up period of six months for evaluation of MACE and clinically significant bleeding. Studies focusing exclusively on non-PCI populations, lacking a direct comparison between DAPT and monotherapy, or presenting incomplete outcome data were excluded.

Additional exclusion criteria included studies involving outdated antiplatelet regimens, pediatric populations, or non-English language publications to maintain consistency and relevance. Furthermore, meta-analyses, editorials, and review articles were not included in the systematic review, as they do not provide primary patient-level data. Studies with incomplete data, a high risk of bias, or lacking clear definitions of endpoints were also excluded to ensure the integrity of the findings. When multiple studies reported findings from the same clinical trial or registry, the most recent and comprehensive dataset was selected to avoid duplication and redundancy. The strict adherence to these eligibility criteria ensured that only robust, high-quality evidence contributed to the final analysis, strengthening the validity of the conclusions drawn in this review.

Data Extraction

Data extraction was conducted systematically to ensure accuracy, consistency, and completeness in synthesizing relevant information from the selected studies. A structured data extraction form was used to collect key details, including study design, sample size, patient demographics, intervention and comparator regimens, duration of follow-up, and primary and secondary outcomes. Specific endpoints, such as MACE, bleeding rates (BARC criteria [8]), and all-cause mortality, were carefully recorded. To minimize bias and enhance reliability, two independent reviewers extracted data separately, with discrepancies resolved through discussion or consultation with a third reviewer. In cases where essential information was missing, corresponding authors were contacted, or supplementary materials were reviewed to ensure completeness. Data from intention-to-treat and per-protocol analyses were prioritized, and hazard ratios (HRs), confidence intervals (CIs), and p-values were extracted as reported in the original studies. This structured approach ensured a comprehensive and reproducible data synthesis for the systematic review.

Quality Assessment

To evaluate the methodological rigor of the included studies, a formal quality assessment was conducted. RCTs were assessed using the Cochrane Risk of Bias 2.0 (RoB 2.0) tool, which examines key domains including random sequence generation, allocation concealment, blinding of participants and outcome assessors, completeness of outcome data, and selective reporting. Post-hoc analyses were evaluated using the Newcastle-Ottawa Scale (NOS), focusing on selection, comparability, and outcome measures. Two reviewers independently conducted the assessments, and any discrepancies were resolved through discussion or adjudication by a third reviewer. Studies were categorized as having low, moderate, or high risk of bias based on their individual domain scores. This process ensured the inclusion of high-quality evidence and enhanced the reliability of the review's conclusions.

Data Analysis and Synthesis

Data analysis and synthesis were conducted in accordance with PRISMA guidelines to ensure methodological rigor and transparency. Extracted data were systematically organized and analyzed to compare the efficacy and safety of different antiplatelet therapy regimens following PCI. The primary endpoints, including MACE and bleeding rates, were assessed across studies, with HRs and CIs extracted to facilitate direct comparison. A qualitative narrative synthesis was performed to summarize key findings, highlighting consistencies and discrepancies between studies. Statistical heterogeneity among trials was considered, and where applicable, subgroup analyses were examined to explore variations in treatment effects based on patient demographics and clinical characteristics. Sensitivity analyses were also reviewed to assess the robustness of results. The synthesis aimed to provide a comprehensive, evidence-based evaluation of the potential benefits and risks of transitioning to monotherapy after dual antiplatelet therapy in high-risk patients who underwent PCI.

Results

Study Selection Process

**Figure 1:**
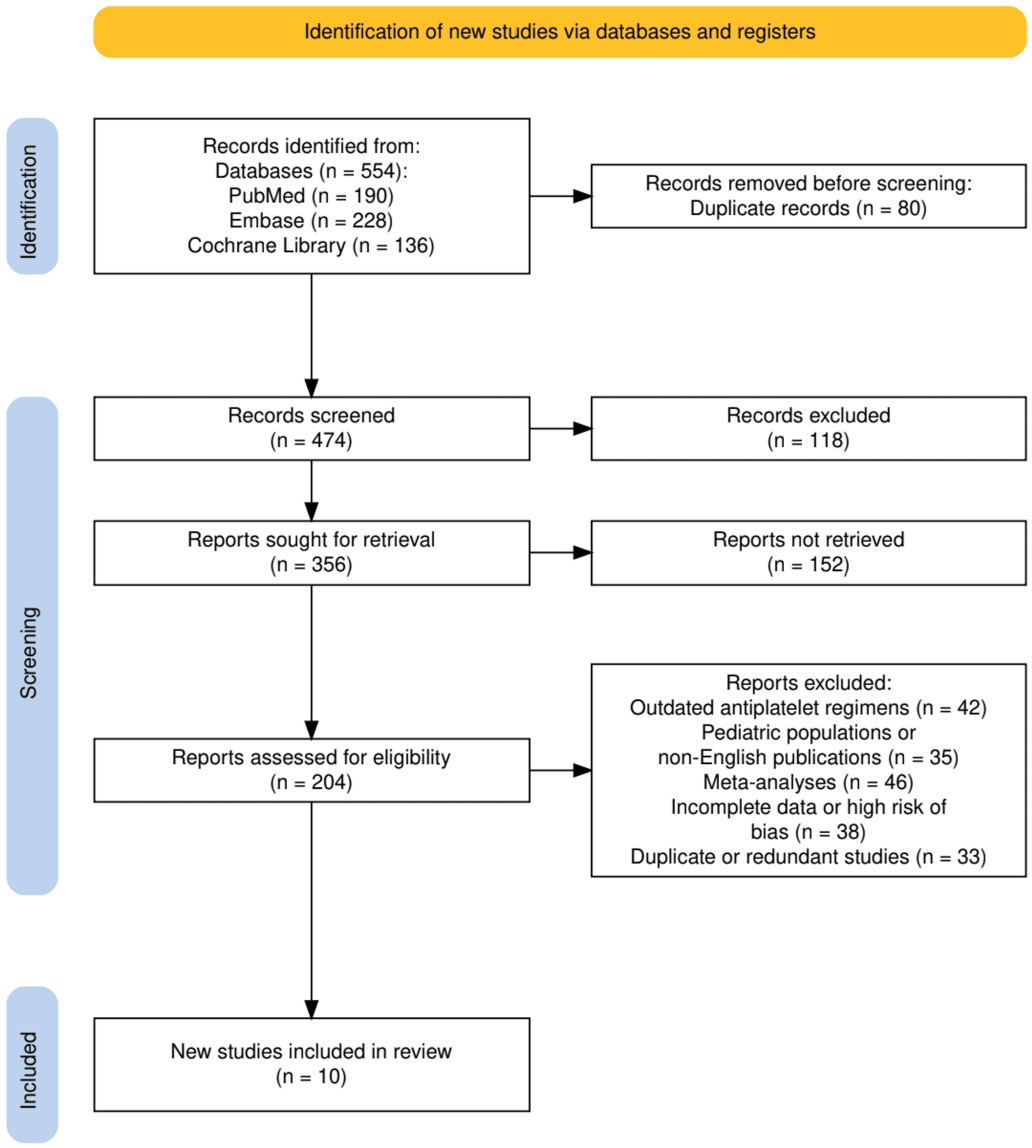
The PRISMA flowchart representing the study selection process PRISMA: Preferred Reporting Items for Systematic Reviews and Meta-Analyses; EMBASE: Excerpta Medica database

The study selection process adhered to a rigorous and systematic approach to ensure the inclusion of high-quality evidence. As illustrated in Figure 1, a total of 554 records were initially identified from three major databases: PubMed (190 records), EMBASE (228 records), and Cochrane Library (136 records). Following the removal of 80 duplicate records, 474 unique studies proceeded to the screening stage. At this stage, 118 studies were excluded based on title and abstract review due to irrelevance to the research question. Subsequently, 356 full-text reports were sought for retrieval; however, 152 reports could not be retrieved, leaving 204 studies for full-text eligibility assessment. After a detailed review, 94 reports were further excluded based on predefined exclusion criteria, including outdated antiplatelet regimens (42 studies), pediatric populations or non-English publications (35 studies), incomplete data or high risk of bias (38 studies), and duplicate or redundant studies (33 studies). Ultimately, 10 studies met the eligibility criteria and were included in the final systematic review. This multi-step selection process, in accordance with PRISMA guidelines, ensured that the review was based on high-quality, relevant, and methodologically sound studies.

Characteristics of the Selected Studies

The characteristics of the selected studies are summarized in Table 1, which includes 10 RCTs and post-hoc analyses assessing the efficacy and safety of P2Y12 inhibitor monotherapy compared to DAPT after PCI. The sample sizes varied, ranging from 1,387 to 11,289 patients, with diverse populations including ACS patients, high bleeding risk cohorts, and stable post-PCI patients. The mean ages of participants ranged from 61 to 71.6 years, with varying proportions of female participants (20%-22.3%) and ST-elevation myocardial infarction (STEMI)/non-STEMI (NSTEMI) cases. The intervention arms predominantly featured ticagrelor or clopidogrel monotherapy following an initial period of DAPT, with durations of monotherapy ranging from one to 11 months. The primary outcomes assessed across the studies included MACE, major bleeding (BARC type 2, 3, or 5 [8]), MI, stroke, and stent thrombosis. Notably, several studies demonstrated a significant reduction in bleeding risk with monotherapy compared to prolonged DAPT, while cardiovascular event rates remained similar across groups. These findings highlight the potential benefit of P2Y12 inhibitor monotherapy in reducing bleeding complications while maintaining ischemic protection, thereby supporting its use as an alternative strategy in post-PCI patients.

**Table 1:** Characteristics of the selected studies RCT: randomized controlled trial; ACS: acute coronary syndrome; PCI: percutaneous coronary intervention; DAPT: dual antiplatelet therapy; BID: bis in die (twice a day); OD: omni die (once a day); CV: cardiovascular; MI: myocardial infarction; STEMI: ST-elevation myocardial infarction; NSTEMI: non-ST-elevation myocardial infarction; MACCE: major adverse cardiac and cerebrovascular events; MACE: major adverse cardiovascular events; HR: hazard ratio; CI: confidence interval; BARC: Bleeding Academic Research Consortium; P2Y12: platelet P2Y12 receptor inhibitor (e.g., clopidogrel, prasugrel, ticagrelor)

Study	Design	Population (N, Age, Risk Factors)	Intervention (DAPT Regimen, Duration)	Comparison (Monotherapy, Duration)	Primary Outcomes	MACE Rate (% or HR, CI)	Bleeding Rate (% or HR, CI)
Ge et al., 2024 [10]	RCT (double-blind, placebo-controlled)	N=3,400, ACS patients, event-free at 1 month post PCI	Ticagrelor (90 mg BID) + aspirin (100 mg OD) for 12 months	Ticagrelor (90 mg BID) alone for 11 months	Clinically relevant bleeding (BARC 2,3,5), MACCE	3.6% ticagrelor vs. 3.7% DAPT; HR 0.98 (95% CI 0.69-1.39)	2.1% ticagrelor vs. 4.6% DAPT; HR 0.45 (95% CI 0.30-0.66)
Watanabe et al., 2022 [11]	RCT (multicenter, open-label)	N=4,136, ACS patients, Mean age 66.8 years, 21% female, 56% STEMI, 20% NSTEMI	Clopidogrel (75 mg OD) + aspirin (100 mg OD) for 12 months	Clopidogrel (75 mg OD) alone after 1-2 months of DAPT	Composite of CV death, MI, stroke, stent thrombosis, bleeding	3.2% clopidogrel vs. 2.8% DAPT; HR 1.14 (95% CI 0.80-1.62)	0.5% clopidogrel vs. 1.2% DAPT; HR 0.46 (95% CI 0.23-0.94)
Watanabe et al., 2024 [12]	RCT (multicenter, open-label, adjudicator-blinded)	N=3,005, Mean age 68.6 years, 38.3% ACS, 22.3% female	Clopidogrel (75 mg OD) + aspirin (100 mg OD) for 1 month, then clopidogrel alone for 5 years	Aspirin (100 mg OD) alone after 12 months of DAPT	Composite of CV death, MI, stroke, stent thrombosis, major bleeding	11.75% clopidogrel vs. 13.57% aspirin; HR 0.85 (95% CI 0.70-1.05)	4.44% clopidogrel vs. 4.92% aspirin; HR 0.89 (95% CI 0.64-1.25)
Natsuaki et al., 2024 [13]	RCT (multicenter, open-label)	N=5,966, ACS (75%) or high bleeding risk, Mean age 71.6 years, 76.6% male	Prasugrel (3.75 mg/day) + aspirin (81-100 mg/day) for 1 month	Prasugrel (3.75 mg/day) alone for 1 month	Major bleeding (BARC 3, 5), cardiovascular events	4.12% prasugrel vs. 3.69% DAPT; HR 1.12 (95% CI 0.87-1.45)	4.47% prasugrel vs. 4.71% DAPT; HR 0.95 (95% CI 0.75-1.20)
Hong et al., 2024 [14]	RCT (noninferiority, open-label)	N=2,850, ACS, Mean age 61 years, 40% STEMI	Ticagrelor (90 mg BID) + aspirin (100 mg OD) for 12 months	Ticagrelor monotherapy after <1 month of DAPT	Composite of death, MI, stent thrombosis, stroke, major bleeding	2.8% ticagrelor vs. 5.2% DAPT; HR 0.54 (95% CI 0.37-0.80)	1.2% ticagrelor vs. 3.4% DAPT; HR 0.35 (95% CI 0.20-0.61)
Kim et al., 2020 [15]	RCT (multicenter)	N=3,056, ACS, Mean age 61 years, 20% female	Ticagrelor (90 mg BID) + aspirin (100 mg OD) for 12 months	Ticagrelor monotherapy after 3 months of DAPT	Major bleeding and adverse cardiac/cerebrovascular events	3.9% ticagrelor vs. 5.9% DAPT; HR 0.66 (95% CI 0.48-0.92)	1.7% ticagrelor vs. 3.0% DAPT; HR 0.56 (95% CI 0.34-0.91)
Min et al., 2024 [16]	RCT (noninferiority, open-label)	N=1,387, Mean age 63.0 years, 76.1% male	P2Y12 inhibitor + aspirin for 3 months	P2Y12 inhibitor monotherapy after 3 months of DAPT	Net adverse clinical event (major bleeding + MACCE)	1.7% P2Y12 vs. 2.6% DAPT; HR 0.93 (95% CI 0.77-1.20)	0.2% P2Y12 vs. 0.8% DAPT; HR 0.60 (95% CI 0.33-1.11)
Yang et al., 2023 [17]	Post-hoc analysis of RCT	N=5,403, event-free for 6-18 months post PCI	Clopidogrel vs. aspirin monotherapy after PCI	Clopidogrel vs. aspirin monotherapy	All-cause death, MI, stroke, ACS readmission, major bleeding	Clopidogrel superior to aspirin; HR 0.73 (95% CI 0.59-0.90)	Clopidogrel superior to aspirin; HR 0.73 (95% CI 0.59-0.90)
Chichareon et al., 2020 [18]	Post-hoc analysis of RCT	N=11,289, event-free at 12 months post PCI, stratified by DAPT score	Ticagrelor monotherapy vs. aspirin monotherapy in the second year post PCI	Ticagrelor vs. aspirin monotherapy in the second year post PCI	Ischemic events (MI, stent thrombosis) and major bleeding	0.70% low DAPT vs. 1.55% high DAPT; p < 0.0001	0.54% low DAPT vs. 0.30% high DAPT; p = 0.058
Mehran et al., 2019 [19]	RCT (double-blind)	N=9006, High-risk PCI patients, event-free at 3 months post PCI	Ticagrelor (90 mg BID) + aspirin (100 mg OD) for 12 months	Ticagrelor (90 mg BID) alone for 9 months	BARC 2,3,5 bleeding; death, MI, stroke	3.9% ticagrelor vs. 3.9% DAPT; HR 0.99 (95% CI 0.78-1.25)	4.0% ticagrelor vs. 7.1% DAPT; HR 0.56 (95% CI 0.45-0.68)

Quality Assessment

The quality assessment of the included studies revealed varying levels of risk of bias, as summarized in Table 2. Double-blind, placebo-controlled RCTs demonstrated low risk of bias across all domains, with proper random sequence generation, allocation concealment, and blinding of participants and outcome assessment. In contrast, open-label studies exhibited a moderate risk of bias, primarily due to a lack of blinding in participant and personnel assessment, which could introduce performance bias. However, these studies ensured low attrition and reporting bias, maintaining their overall validity. Post-hoc analyses were assessed using the NOS [20], scoring 7/9, indicating good quality but with potential concerns due to unclear handling of incomplete data. Despite some methodological limitations, particularly regarding blinding, the rigorous randomization and comprehensive data reporting across studies support the reliability of the findings in this systematic review.

**Table 2:** The quality assessment of the included studies RCT: randomized controlled trial

Study	Design	Random Sequence Generation (Selection Bias)	Allocation Concealment (Selection Bias)	Blinding of Participants & Personnel (Performance Bias)	Blinding of Outcome Assessment (Detection Bias)	Incomplete Outcome Data (Attrition Bias)	Selective Reporting (Reporting Bias)	Overall Risk of Bias (RoB) or Newcastle-Ottawa Scale (NOS) Score
Ge et al., 2024 [10]	RCT (double-blind, placebo-controlled)	Low	Low	Low	Low	Low	Low	Low risk
Watanabe et al., 2022 [11]	RCT (multicenter, open-label)	Low	Unclear	High (open-label)	Low	Low	Low	Moderate risk
Watanabe et al., 2024 [12]	RCT (multicenter, open-label, adjudicator-blinded)	Low	Low	High (open-label)	Low	Low	Low	Moderate risk
Natsuaki et al., 2024 [13]	RCT (multicenter, open-label)	Low	Low	High (open-label)	Low	Low	Low	Moderate risk
Hong et al., 2024 [14]	RCT (noninferiority, open-label)	Low	Unclear	High (open-label)	Low	Low	Low	Moderate risk
Kim et al., 2020 [15]	RCT (multicenter)	Low	Low	High (open-label)	Low	Low	Low	Moderate risk
Min et al., 2024 [16]	RCT (noninferiority, open-label)	Low	Low	High (open-label)	Low	Low	Low	Moderate risk
Yang et al., 2023 [17]	Post-hoc analysis of RCT	Not applicable	Not applicable	Not applicable	Unclear	Low	Low	NOS: 7/9 (good quality); Selection: 4/4, Comparability: 1/2, Outcome: 2/3
Chichareon et al., 2020 [18]	Post-hoc analysis of RCT	Not applicable	Not applicable	Not applicable	Unclear	Low	Low	NOS: 7/9 (good quality); Selection: 4/4, Comparability: 1/2, Outcome: 2/3
Mehran et al., 2019 [19]	RCT (double-blind)	Low	Low	Low	Low	Low	Low	Low risk

Discussion

The findings of these studies provide substantial evidence supporting the use of P2Y12 inhibitor monotherapy following an initial period of DAPT in patients undergoing PCI. Across multiple randomized controlled trials, ticagrelor or clopidogrel monotherapy consistently demonstrated a significant reduction in bleeding risk without a corresponding increase in major adverse cardiovascular or cerebrovascular events (MACCE). For instance, in the Ultra-Short Dual Antiplatelet Therapy Followed by Ticagrelor Monotherapy After Percutaneous Coronary Intervention in Patients With Acute Coronary Syndromes (ULTIMATE-DAPT) trial [10], ticagrelor monotherapy was associated with a lower rate of clinically relevant bleeding (2.1% vs. 4.6%; HR 0.45, 95% CI 0.30-0.66) compared to ticagrelor plus aspirin, while maintaining similar MACCE rates (3.6% vs. 3.7%). Similar benefits were observed in the Ticagrelor Monotherapy Following Stent Placement in Acute Coronary Syndrome Patients Study (T-PASS) trial [14], where ticagrelor monotherapy after less than one month of DAPT significantly lowered major bleeding risk (1.2% vs. 3.4%; HR 0.35, 95% CI 0.20-0.61) while demonstrating non-inferiority for overall cardiovascular outcomes. The findings from these studies reinforce the potential of early aspirin discontinuation as a safer alternative for high-risk patients, particularly those at an increased risk of bleeding.

Moreover, the comparison between clopidogrel and aspirin monotherapy post DAPT has yielded significant insights. Watanabe et al. [12] reported that clopidogrel monotherapy beyond one month of DAPT was noninferior to aspirin monotherapy in preventing MACCE but demonstrated a trend toward lower cardiovascular event rates (8.61% vs. 11.05%; HR 0.77, 95% CI 0.61-0.97). In a post-hoc analysis [17], clopidogrel also showed superiority over aspirin in reducing both ischemic and bleeding complications (HR 0.73, 95% CI 0.59-0.90). These findings suggest that clopidogrel may offer a more favorable balance between thrombotic and hemorrhagic risks in long-term secondary prevention following PCI. Furthermore, in the Ticagrelor With Aspirin or Alone in High-Risk Patients After Coronary Intervention (TWILIGHT) trial [19], ticagrelor monotherapy significantly reduced BARC 2, 3, and 5 bleeding rates (4.0% vs. 7.1%; HR 0.56, 95% CI 0.45-0.68) without increasing the risk of death, MI, or stroke. Collectively, these studies provide compelling evidence supporting the de-escalation of antiplatelet therapy in select patient populations, highlighting the need for individualized treatment strategies to optimize safety and efficacy.

The findings of this analysis are largely consistent with previous landmark trials, such as the TWILIGHT trial [19] and the Ticagrelor Monotherapy After 3 Months of Dual Antiplatelet Therapy in Acute Coronary Syndrome (TICO) trial [15], which demonstrated that ticagrelor monotherapy following an initial period of DAPT significantly reduces major bleeding events without increasing ischemic risks. In the TWILIGHT trial [19], ticagrelor monotherapy reduced BARC 2,3,5 bleeding rates (4.0% vs. 7.1%; HR 0.56, 95% CI 0.45-0.68) without increasing MACCE, a trend similarly observed in the study by Ge et al. [10] and Hong et al. [14]. Furthermore, the T-PASS trial [14] extended this observation to patients with ACS, where ticagrelor monotherapy after less than one month of DAPT resulted in a significantly lower bleeding rate (1.2% vs. 3.4%; HR 0.35, 95% CI 0.20-0.61) while maintaining non-inferiority in preventing MACCE. This further reinforces the growing body of evidence supporting early aspirin discontinuation as a safer alternative for high-risk patients post PCI.

However, some differences exist between recent studies and prior literature regarding the optimal duration of DAPT before transitioning to monotherapy. The Short and Optimal Duration of Dual Antiplatelet Therapy After Everolimus-Eluting Stent in Acute Coronary Syndrome (STOPDAPT-2 ACS) trial [11] failed to demonstrate non-inferiority for clopidogrel monotherapy after one to two months of DAPT compared to standard 12-month DAPT, primarily due to a numerically higher cardiovascular event rate (3.2% vs. 2.8%; HR 1.14, 95% CI 0.80-1.62). This contrasts with the study by Watanabe et al. [12], which found clopidogrel monotherapy beyond one month to be noninferior to aspirin monotherapy in preventing MACCE, suggesting that the timing of aspirin discontinuation may be critical in determining outcomes. Additionally, the Short Duration of Dual Antiplatelet Therapy Followed by P2Y12 Inhibitor Monotherapy After Drug-Eluting Stent Implantation (SHARE) trial [16] found that P2Y12 inhibitor monotherapy after three months of DAPT was noninferior to 12-month DAPT but emphasized the need for further research in broader populations. These findings collectively indicate that while the de-escalation strategy is effective in reducing bleeding, individual patient risk factors, PCI complexity, and the specific antiplatelet regimen used may influence overall efficacy.

The observed reduction in bleeding risk with P2Y12 inhibitor monotherapy, particularly ticagrelor or clopidogrel, can be attributed to the elimination of aspirin-related bleeding complications while maintaining adequate platelet inhibition [21]. Aspirin, despite its well-established role in secondary prevention, potentiates gastrointestinal mucosal injury and increases the risk of intracranial hemorrhage due to its irreversible inhibition of cyclooxygenase-1 (COX-1), which suppresses thromboxane A2 production and impairs primary hemostasis [22,23]. In contrast, ticagrelor and clopidogrel selectively inhibit the P2Y12 receptor, reducing platelet aggregation while preserving the role of other hemostatic pathways, thereby lowering the risk of major bleeding without significantly increasing ischemic events. The results of Ge et al. [10] and Mehran et al. [19] support this mechanism, as patients who continued ticagrelor alone after an initial period of DAPT exhibited a significantly lower bleeding rate without an increase in MACCE. Additionally, the pharmacokinetics of ticagrelor, which provides rapid and reversible inhibition of platelet activation, may contribute to a more controlled and predictable antithrombotic effect, further enhancing safety in high-risk patients [24]. These findings have significant clinical implications, particularly for patients at high risk of bleeding, suggesting that early aspirin discontinuation may be a viable strategy to optimize long-term antiplatelet therapy following PCI while maintaining adequate ischemic protection [25].

This study possesses several key strengths that enhance its validity and reliability. The large sample size across multiple trials, particularly in studies like Ge et al. [10] and Mehran et al. [19], ensures sufficient statistical power to detect significant differences between ticagrelor monotherapy and DAPT. The RCT designs of these studies minimize selection bias and allow for a balanced comparison between intervention and control groups. Additionally, trials such as Watanabe et al. [11] implemented blinding and allocation concealment, reducing the risk of performance and detection bias. The inclusion of intention-to-treat analyses and robust statistical methods further strengthens the credibility of the results, ensuring that findings are both clinically and statistically meaningful. However, despite these strengths, several limitations must be acknowledged. Many of the studies had a relatively short follow-up duration, with most focusing on outcomes within 12 months post PCI, limiting the ability to assess long-term safety and efficacy of P2Y12 monotherapy beyond one year. Additionally, certain trials, such as Hong et al. [12], were open-label, which introduces a risk of bias in reporting subjective outcomes like bleeding events. Furthermore, differences in patient populations, including varying proportions of STEMI, NSTEMI, and high bleeding risk patients, may limit the generalizability of findings to all PCI patients. Finally, as many studies were conducted in specific geographic regions, particularly East Asia, the results may not fully translate to Western populations with differing genetic and lifestyle factors influencing drug metabolism and cardiovascular risk profiles.

The findings of this study have important clinical and practical implications for the management of patients undergoing PCI. The consistent reduction in clinically significant bleeding with P2Y12 inhibitor monotherapy, as observed in studies like Ge et al. [10] and Mehran et al. [19], suggests that early aspirin discontinuation after an initial period of DAPT could be a safer alternative for high-risk patients without compromising cardiovascular protection. Given that major bleeding events are associated with higher morbidity and mortality, adopting ticagrelor or clopidogrel monotherapy in appropriately selected patients may improve long-term outcomes. These results align with emerging evidence from prior trials like TWILIGHT, reinforcing the potential shift in antiplatelet therapy guidelines. Clinicians should consider individualizing treatment strategies, particularly for patients with high bleeding risk, while ensuring adequate ischemic protection in those at higher thrombotic risk.

Future research should focus on long-term follow-up studies to assess the durability of benefits associated with P2Y12 inhibitor monotherapy beyond the typical 12-month post-PCI period. While existing trials, including Ge et al. (2024) [10] and Mehran et al. (2019) [19], have demonstrated a reduction in bleeding risk without compromising ischemic protection, the long-term effects on stent thrombosis, late MI, and all-cause mortality remain unclear. Additionally, subgroup analyses are needed to determine which patient populations, such as those with diabetes, chronic kidney disease, or prior stroke, would derive the greatest benefit from early aspirin discontinuation. Comparative studies evaluating newer antiplatelet agents, such as cangrelor or ticagrelor, reversibly binding formulations, may provide further insights into optimizing post-PCI antithrombotic therapy. Furthermore, real-world observational studies and registries could help validate these findings in diverse patient populations and different healthcare settings, ensuring that emerging treatment strategies are both effective and widely applicable.

## Conclusions

This study supports the transition to P2Y12 inhibitor monotherapy, primarily ticagrelor or clopidogrel, after an initial period of DAPT in patients post PCI, particularly those at high risk for bleeding. The findings demonstrate that monotherapy significantly reduces clinically relevant bleeding events without increasing the incidence of MACCE. These results reinforce the growing trend toward personalized antithrombotic strategies that prioritize a balance between safety and efficacy. Most included trials had follow-up durations of up to 12 months, and further long-term studies are warranted to evaluate the durability of these benefits beyond this timeframe. It is also important to note that outcomes may vary depending on patient-specific factors (e.g., bleeding risk, ACS status) and the choice of antiplatelet agent. Additionally, as several trials were conducted predominantly in East Asian populations, caution is advised in generalizing these findings across diverse global populations and healthcare settings. Nonetheless, early aspirin discontinuation in selected patients may represent a safe and effective strategy in contemporary post-PCI management.
